# The evolutionary genetics of paternal care: How good genes and extrapair copulation affect the trade‐off between paternal care and mating success

**DOI:** 10.1002/ece3.7058

**Published:** 2021-01-12

**Authors:** Courtney Fitzpatrick, Colette M. Ciresi, Michael J. Wade

**Affiliations:** ^1^ Department of Biology Indiana University Bloomington IN USA

**Keywords:** direct genetic effects, extrapair copulation, Indirect genetic effects, mathematical model, paternal care

## Abstract

We investigate the evolution of a gene for paternal care, with pleiotropic effects on male mating fitness and offspring viability, with and without extrapair copulations (EPCs). We develop a population genetic model to examine how pleiotropic effects of a male mating advantage and paternal care are affected by “good genes” and EPCs. Using this approach, we show that the relative effects of each on fitness do not always predict the evolutionary change. We then find the line of combinations of mating success and paternal care that bisects the plane of possible values into regions of positive or negative gene frequency change. This line shifts when either good genes or EPCs are introduced, thereby expanding or contracting the region of positive gene frequency change and significantly affecting the evolution of paternal care. Predictably, a direct viability effect of “good genes” that enhances offspring viability constrains or expands the parameter space over which paternal care can evolve, depending on whether the viability effect is associated with the paternal care allele or not. In either case, the effect of a “good gene” that enhances offspring viability is substantial; when strong enough, it can even facilitate the evolution of *poor* paternal care, where males harm their young. When nonrandom mating is followed by random EPCs, the genetic regression between sire and offspring is reduced and, consequently, the relative strengths of selection are skewed away from paternal care and toward the male mating advantage. However, when random mating is followed by nonrandom EPCs, a situation called “trading up” by females, we show that selection is skewed in the opposite direction, away from male mating advantage and toward paternal care across the natural range of EPC frequencies.

## Graphical abstract

We present a population genetic model to investigate the evolution of a gene for paternal care, when it also has pleiotropic effects on male mating fitness and offspring viability, with and without extrapair copulations (EPCs). We illustrate the quantitative evolutionary relationship between the different direct and indirect fitness effects hypothesized to influence the evolution of male parental care. We model trade‐offs: (1) between a direct effect on male mating success and an indirect effect on paternal caregiving; (2) between male mating success and offspring viability; and, (3) between good genes and bad parenting or vice versa. We show that a male mating bias is an episode of selection occurring before reproduction and offspring viability selection. Moreover, it differentially affects the direct and indirect components of offspring viability selection as they affect mean fitness and gene frequency change.

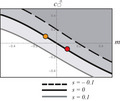

## 1 INTRODUCTION

The evolution of paternal offspring care in nonmonogamous mating systems is believed to be difficult when it occurs at the expense of additional mating opportunities and at the risk of extrapair copulations (EPCs) by females (Magrath & Komdeur, 2003; McGlothlin et al., 2007). The first of these obstacles, a trade‐off between mate seeking and offspring care by males, is considered by many to be the most significant impediment to the evolution of paternal care because additional mates are believed generally to provide a larger fitness increment than the “relative value” of paternal care (Clutton‐Brock, 1991; Maynard‐Smith, 1977; Webster, 1991 but see Queller, 1997; Wade & Shuster, 2002). The assumption that such a trade‐off impedes the evolution of paternal care has been reinforced because empirical studies, which have found evidence for the trade‐off itself across a wide range of taxa (e.g., birds, Mitchell et al., 2007; mammals, Smorkatcheva et al., 2010; spiders, Alissa et al., 2017).

Many view EPCs by females as a second major impediment to the evolution of paternal care because, with EPCs, there is lowered paternity assurance. Therefore, a male may end up rearing the genetic offspring of other males, incurring a fitness cost for himself while generating a fitness benefit for another, unrelated individual in the population (Moller, 2000). Nonetheless, EPCs are hypothesized to be adaptive for females for many reasons including obtaining “good mating genes” or other male attributes for sons (Birkhead & Moller, 1992; Heisler 1981; Weatherhead and Robertson 1979), for confusing paternity in order to prevent infanticide (Wolff & MacDonald, 2004), for fertility assurance (Sheldon, 1994), or for securing good viability genes for their offspring (Petrie & Kempenaers, 1998). While it remains controversial which, if any of these hypothesized fitness benefits, are the underlying cause of adaptive female EPCs (Akçay & Roughgarden, 2007; Forstmeier et al., 2014), these hypotheses assume heritable variation among males in mating success, paternal care, and viability and that variation in each predicts variation in fitness.

Although most hypothesize a trade‐off between male mating success and male offspring care, some have hypothesized the opposite, a synergy. Under the “good parent hypothesis” (Buchanan & Catchpole, 2000; Hoelzer, 1989; Linville et al., 1998), male sexual traits may signal paternal care‐giving ability, permitting females to exert a mating preference for care‐giving males. Under this hypothesis, male mating success and male caregiving are positively synergistic rather than in opposition.

Given the many hypotheses, it is difficult a priori to predict how male fitness attributes affect the evolution of male parental care, especially when there is nonrandom mating owing to male mating advantages or to EPCs. We develop a common population genetic framework to evaluate these hypotheses, one that allows a single gene to have pleiotropic effects on fitness. Most models of the evolution of paternal care are game theoretical optimality models, following the seminal work of Houston et al. (2013) and Maynard Smith (1977). However, when genes have direct and transgenerational fitness effects, populations do not necessarily evolve to the peak that maximizes mean fitness in the population (Dury & Wade, 2019; Wolf & Wade, 2016). Moreover, inbreeding, a type of nonrandom mating, has been shown to alter the weightings of direct and indirect effects (Wolf & Wade, 2016)—here, we extend that finding to another kind of nonrandom mating, namely male mating advantage.

Our population genetic framework allows us to model trade‐offs as well as synergies among male mating success, male offspring care, and good genes (Andersson, 2005; Brommer et al., 2007; Curtsinger & Heisler, 1988). We are not proposing that complex behaviors have a simple single‐gene architecture, but rather that studying the dynamics of individual loci lends insight into the underlying causes of quantitative genetic variation and patterns of evolution. Moreover, we can directly compare the relative rates of evolutionary genetic change for different scenarios, which allows us to ask whether a male mating advantage associated with paternal care will evolve faster or slower than one associated with good genes (Moller, 2000; Wade, 2014). In this way, we can untangle some of the evolutionary complexity of the interactions between male mating success, male care, offspring viability, and the frequency of EPCs (Curtsinger & Heisler, 1988; Ketterson & Nolan, 1994). Our findings illuminate the circumstances under which fitness costs to males, such as forgoing mating opportunities, can be offset by fitness gains to offspring through enhanced viability, and we show how EPCs affect these relationships.

## 2 THE MODEL

We assume a simple genetic model where paternal care is determined additively by a single locus with alternative alleles, C and c, with pleiotropic direct and indirect effects on male fitness (cf. models of Wade (1998, 2001) or Wolf and Wade (2016), on maternal effects). Many population genetic models of kin selection (e.g., (Lehmann, 2007; Nowak, 2006; Wade, 1980) are similarly structured postulating alternative alleles at a single locus, each with a direct fitness effect (e.g., cost of altruism to the performing genotypes) and an indirect fitness effect (e.g., benefit of altruism to the receiving genotypes). Genomic studies in laying hens provide empirical support for the existence of single genes with such pleiotropic direct and indirect effects on viability and feather quality, respectively (Biscarini et al., 2010; Fan et al., 2013). The latter is particularly relevant to our model because feather quality has been associated with male mating advantage in birds (Hamilton & Zuk, 1982; Shuster & Wade, 2003; Wade, 2014).

### 2.1 Offspring viability affected both by offspring genotype and by paternal genotype

We first investigate direct and indirect effects on offspring viability and male parental care, before introducing either a male mating advantage or EPCs. In population genetics, an individual experiences a direct effect of a gene when that gene resides in the individual's own genome and an individual experiences an indirect effect of a gene when that gene resides in the genome of a different individual. Our model examines the potential for evolution of an allele that has both direct effects (via survival) and indirect effects (via paternal care).

We associate these direct and indirect fitness effects with particular genotypes in the following ways. We let the c allele have an additive, *direct effect*, *s*, on viability in both males and females; individuals of genotype Cc have enhanced viability by the increment *s*, relative to individuals with the CC genotype. Similarly, cc individuals have enhanced viability by 2s. In addition to this direct effect, we model paternal care by letting the c allele in males have an *indirect effect* on the survival of their offspring; males of genotype cc increment the viability fitness of their offspring by the quantity 2c_♂_, while Cc heterozygous males increment the fitness of their offspring by c_♂,_ half as much. Again, these offspring viability benefits are relative to the CC genotype, which provides no paternal care. As a result of these direct and indirect genetic effects, Cc offspring of cc fathers have total viability fitness, 1 + *s* + 2c_♂,_ while the viability of cc offspring of Cc fathers is different, namely 1 + 2*s* + c_♂_. In other words, the “c” allele acts in adult males and causes them to care for (c_♂_ > 0) or harm (c_♂_ < 0) their offspring; and, the same “c” allele in an offspring causes it to live a longer life (*s* > 0) or a shorter (*s* < 0) life.

We now compare how the direct (s) and indirect (c_♂_) fitness effects on offspring viability affect the evolution of the c allele, first where males and females mate randomly and each has one mate. After that, we will introduce male mating effects such as those that might result from female choice and male–male competition or from extrapair copulations, and examine how they further impact the evolutionary trajectory of the “c” allele. Because we will be introducing nonrandom mating and because care‐giving male genotypes experience differential viability during their own development prior to mating, we do not assume that the genotypes of breeding parents are in Hardy–Weinberg proportions. Instead, we let the frequency of CC males and females be P, Cc heterozygotes be H, and cc homozygotes be Q, noting that (P + H + Q) = 1 in each sex. With random mating among these genotypes, we have the entries in Table 1. Under these assumptions, mean offspring viability fitness, *W*, equals.

(1) $$W=1+2q\left(s+c_{♂}\right),$$

where *q*, the frequency of the c allele, is ([H/2] + Q). Note that the direct effect, *s*, and indirect effect, c_♂_, on offspring viability are weighted equally (by 2*q*) in the mean fitness function, *W*. Thus, a 10% increase in offspring viability owing to the c allele's direct viability effect (*s* = 0.10) increases mean fitness, W, just as much as a 10% increase in offspring viability owing to the c allele's effect on paternal care (c_♂_ = 0.10).

**TABLE 1:** Monogamous families with male parental care

Male	Female	Family frequency	Offspring genotypes			Family mean fitness
			CC	Cc	cc	
CC	CC	P^2^	1	–	–	1
CC	Cc	PH	1/2	1/2	–	1 + *s*/2
CC	cc	PQ	–	1	–	1 + *s*
Cc	CC	HP	1/2	1/2	–	1 + *s*/2 + 1c_♂_
Cc	Cc	H^2^	1/4	1/2	1/4	1 + *s* + 1c_♂_
Cc	cc	HQ	–	1/2	1/2	1 + 3*s*/2 + 1c_♂_
cc	CC	QP	–	1	–	1 + *s* + 2c_♂_
cc	Cc	QH	–	1/2	1/2	1 + 3*s*/2 + 2c_♂_
cc	cc	Q^2^	–	–	1	1 + 2*s* + 2c_♂_

After viability selection, *P*′, the frequency of CC homozygous adult offspring, is {*p* ^2^ + c_♂_[H*p*/2]}/*W*; the frequency of Cc adult offspring, *H*′, is {2*pq*[1 + *s*] + c_♂_[*q*H/2 + *pq* + Q*p*]}/*W*; and, that of cc adult offspring, *Q*′, is (*q* ^2^[1 + 2*s*] + c_♂_[*q* ^2^ + Q*q*]}/*W*. From these genotype frequencies, we calculate the frequency of the c allele, *q*′, as (*Q*′ + *H*′/2) and obtain (q + *s*[q + q^2^] + c_♂_[pq/2 + Qp/2 + Hq/4])/*W*. Thus, the change in allele frequency in one generation equals.

(2) $$\Delta q=\left\{spq+c_{♂}\left[\left(pq/2\right)‐\left(q^{2}/2\right)+Q/2\right]\right\}/W.$$

If selection is weak, then Q ~ q^2^, allowing us to simplify Equation (2) to

(3) $$\Delta q∼\left\{\left(s+\left[c_{♂}/2\right]\right)pq\right\}/W.$$

Note that, although the direct and indirect fitness effects are weighted equally in the calculation of mean fitness, *W*, (see Equation 1), they are weighted *unequally* when fitness is translated to allele frequency change (see Equation 3). The direct viability effect is weighted by 1 because it is expressed in the offspring's own genome. In contrast, the indirect effect of paternal care is weighted by ½, as in kin selection models, accounting for the genetic regression of offspring on male parent. As a result, a 10% increase in offspring viability from a direct viability effect (*s* = 0.10) has a twice larger effect on the change in the c allele's frequency as a 10% increase in offspring viability from paternal care (c_♂_ = 0.10).

## 3 PATERNAL CARE WITH NONRANDOM MATING

### 3.1 Nonrandom mating

To model nonrandom mating, we define the mating fitness of CC males as 1, Cc males as (1 + *m*) and cc males as (1 + 2*m*). Our parameter, m, allows us to bias mating either toward cc males, when *m* > 0, or toward CC males, when *m* < 0. We do not specify the cause of m, the male mating advantage. It could be owing to female mate choice, where mating females share a preference for (against) some male genotypes over others (as in Kokko et al. 2003). Alternatively, our model can represent a system in which females do not exhibit preferences, but where male–male competition for mates results in males of certain genotypes mating more frequently than expected by chance because they win contests with other males.

This nonrandom mating (*m* ≠ 0) changes gene frequencies in the pool of mating parents and in the offspring derived from them, introducing an episode of selection prior to viability selection. That is, nonrandom mating means that there are two sequential episodes of selection, each changing allele frequency. The first is caused by the male mating advantage; it changes the allele frequency in mating males and therefore in their offspring. It also creates a sex difference in allele frequency. Both selection episodes change allele frequencies, but the magnitude and direction of the changes need not be the same.

With the model above, the average male mating fitness, *W_m_*, is (1 + 2*qm*). And, the frequency of mating CC males becomes *P*′, equal to P times w_CC_, the relative mating fitness of CC males; w_CC_ equals (1/*W_m_*). The frequency of mating Cc males is *H*′ or (Hw_Cc_) with w_Cc_, the relative mating fitness of Cc males, equal to ([1 + *m*]/*W_m_*). The frequency of mating cc males is *Q*′ or *Q*([1 + 2*m*]/*W_m_*). Postmating but before reproduction, the frequency of the c allele in mating males, q_♂_, is (*Q*′ + *H*′/2) and not *q* (i.e., Q + H/2), the allele frequency in females. For clarity, we define q_♀_, as equal to *q* (i.e., Q + H/2). The magnitude of q_♂_, after mating, equals (q_♀_ + Δ*q_m_*), where Δ*q*_m_ = *m*(p_♀_q_♀_ + [Q − *q* ^2^_]_)/*W_m_*. Whenever *m* ≠ 0, nonrandom mating with respect to male genotype results in a sex difference in the gene frequency of male and female parents (q_♂_ ≠ q_♀_ = *q*). The frequency of the “c” allele in the pool of mating parents, *q*′, equals the average across the sexes, (q_♂_ + q_♀_)/2 = (*q* + Δ*q*_m_/2). When there are no EPCs, it is this frequency in the parents that is transmitted to the offspring before viability selection.

The first episode of selection changes the frequency of the c allele in mating pairs, and, for this reason, it changes mean fitness which is a function of the frequency of the c allele at the second episode of selection. This is seen in Equations (4a–c) below where mean viability fitness of offspring after nonrandom mating, W_NRM_, equals.

(4a) $$W_{\text{NRM}}=1+s\left(q_{♀}+q_{♂}\right)+2c_{♂}q_{♂}.$$

(4b) $$W_{\text{NRM}}=1+s\left(2q+\Delta q_{m}\right)+c_{♂}\left(2q+2\Delta q_{m}\right),$$

(4c) $$W_{\text{NRM}}=1+2q\left(s+c_{♂}\right)+\left(\Delta q_{m}\right)\left(s+2c_{♂}\right).$$

Note, first, that, when *m* = 0, then Δ*q_m_* = 0, and Equation (4c) reduces to Equation (1) derived assuming random mating. Note further, comparing Equation (4c) and Equation (1), that nonrandom mating by males changes the coefficients (the weightings) of the direct (*s*) and indirect effects (c_♂_) on mean offspring viability fitness, *W*_NRM_. Subtracting mean fitness, *W*_RM_, for the random mating case from *W*_NRM_ quantifies the effect of the first episode of selection on mean fitness at the second:

(5) $$W_{\text{NRM}}‐W_{\text{RM}}=\left(\Delta q_{m}\right)\left(s+2c_{♂}\right).$$

When mating is random, *m* and Δ*q_m_* are zero, Equation (5) must also be zero. We see from Equation (5) that the indirect effect of paternal care (c_♂_) is affected *twice as heavily* as the direct effect on offspring viability (*s*). The direct effect of the c allele depends on its transmission through dams in frequency q_♀_ and through sires in frequency q_♂_; but, *only* the latter is affected by the nonrandom mating. Differently put, *the direct and indirect effects of the c allele on offspring viability which were weighted equally in W with random mating* (Equation 1) *now are differentially weighted in W*_NRM_ (Equation 4c). The introduction of a male mating advantage complicates the evolutionary trajectory of the c allele.

We calculate *P*″, *H*″, and *Q*″, the genotype frequencies of offspring *after* mating and *after* viability selection through both c_♂_, the indirect effect of paternal care and *s*, the direct effect. We find

(6a) $$P^{\prime \prime }W_{\text{NRM}}=p_{♂}p_{♀}+c_{♂}\left(p_{♀}H^{\prime }/2\right)$$

(6b) $$H^{\prime \prime }W_{\text{NRM}}=\left(1+s\right)\left(p_{♂}q_{♀}+p_{♀}q_{♂}\right)+c_{♂}\left(p_{♀}q_{♂}+\left[q_{♀}H^{\prime }/2\right]+p_{♀}Q^{\prime }\right)$$

and

(6c) $$Q^{\prime \prime }W_{\text{NRM}}=\left(1+2s+c_{♀}\right)\left(q_{♂}q_{♀}\right)+c_{♀}\left(q_{♀}Q^{\prime }\right)$$

where the sum of Equations ((6a), (6b), (6c)) equals Equation (4a), *W*_NRM_ = (1 + *s*[q_♂_ + q_♀_] + c_♂_ [2q_♂_]) = 1 + *s*(2*q* + Δ*q_m_*) + c_♂_ (2q + 2Δ*qm*).

Using Equations ((6a), (6b), (6c)), we then calculate *q*″ from (*Q*″ + *H*″/2) and obtain.

(7) $$q^{\prime \prime }=\left\{\left[q_{♂}+q_{♀}\right]/2+s\left(q_{♂}q_{♀}+\left[q_{♂}+q_{♀}\right]/2\right)+c_{♂}\left(q_{♂}q_{♀}+q_{♂}‐\left[H^{\prime }/4\right]\right)\right\}/W_{\text{NRM}}$$

Subtracting *q*′ equal to [q_♂_ + q_♀_]/2 from *q*″, we have

(8a) $$\Delta \text{qviability}=\left\{s\left(p^{\prime }q^{\prime }‐{\left[\Delta q_{m}/2\right]}^{2}\right)+c_{♂}\left(p_{♂}q_{♂}‐\left[H^{\prime }/4\right]\right)\right\}/W_{\text{NRM}}$$

(8b) $$\Delta \text{qviability}=\left\{s\left(p^{\prime }q^{\prime }‐{\left(\left[q_{♂}‐q_{♀}\right]/2\right)}^{2}\right)+c_{♂}\left(p_{♂}q_{♂}‐\left[H^{\prime }/4\right]\right)\right\}/W_{\text{NRM}}.$$

We can rewrite *p*′*q*′ as (*p* – [Δ*q_m_*/2])(*q* + [Δ*q_m_*/2]) and expand to (*pq* + [*p* – *q*][Δ*q_m_*/2] + [Δ*q_m_*/2]^2^). If that mating advantage and viability selection are so weak that we can discard terms of order (sm), then *s*(*p*′*q*′) reduces to *spq*. Similarly, p_♂_q_♂_ can be rewritten as (*p* – Δ*q_m_*)(*q* + Δ*q_m_*) and expanded to (*pq* + [*p* − *q*][Δ*q_m_*] + [Δ*q_m_*]^2^). With weak selection, *H*′ is ~2*pq*, where the approximation differs from the exact value by less than *m* ^2^ or *s* ^2^. (This difference is so small that the approximate and exact lines overlap and cannot be distinguished in our Figures. For that reason, in all figures, we used the approximate formulas.) With this weak selection approximation, c_♂_(p_♂_q_♂_ − [*H*′/4]) reduces to c_♂_ (*pq*/2). The weak selection assumption allows us to more easily see that the second episode of viability selection here is analogous to the random mating case (see Equation 3 above),

(9) $$\Delta \text{qviability}∼pq\left(s+\left[c_{♂}2\right]\right)/W_{\text{NRM}}.$$

The total frequency change can be expressed as the sum of mating selection followed by viability selection. It is approximately.

(10a) $$\Delta q_{\text{total}}=\left(q^{\prime \prime }‐q\right)=\left(q^{\prime \prime }‐q^{\prime }\right)+\left(q^{\prime }‐q\right).$$

(10b) $$\Delta q_{\text{total}}∼\left(\mathrm{pq}\right)\left\{s+\left[c_{♂}/2\right]\right\}/W_{\text{NRM}}+\left(\mathrm{pq}\right)\left(m/2W_{m}\right).$$

With a parameter for mating bias now introduced, our model is highly flexible and can represent many different biological scenarios within one framework. For instance, we can model a male mating advantage for males with good genes (*s*, *m* > 0). Similarly, we can model a female preference for care‐giving males (c_♂_, *m* > 0) or female avoidance of males who provide poor care or harm (c_♂_, *m* < 0 = *s*). When the effects are of the same sign, the evolutionary fate of the allele of interest is predictable. However, the flexibility of our model lets us investigate situations where two of the three fitness effects oppose each other. We examine some cases below.

### 3.2 Relative strengths of selection via mating bias

**FIGURE 1:**
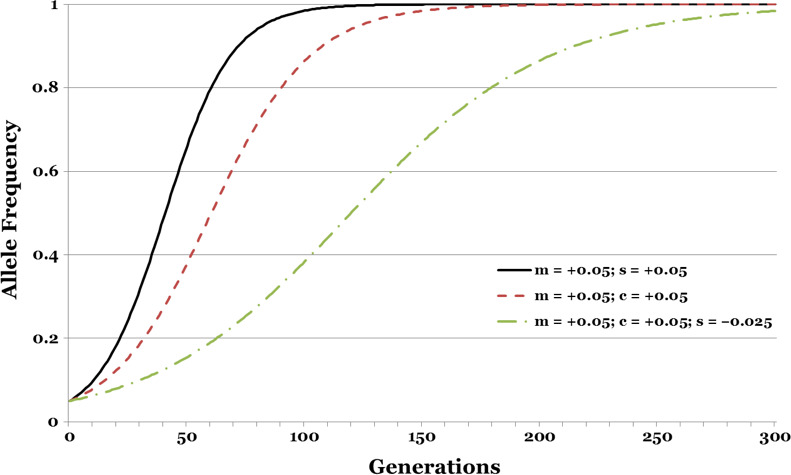
Contrast between (1) a male mating advantage for good genes; (2) a male mating advantage for good parenting; and (3) a male mating advantage for good parenting despite bad genes. With the male mating advantage, mean fitness, W, in the good genes case (1) is *less than* W in the good parenting case (2). Nevertheless, the rate of evolution is *faster* in case (1) than it is in case (2). See text for further discussion

We can first ask, which kind of selection is stronger: a mating bias favoring a good gene or one favoring good paternal care? Remember that, as shown by Equation (5), mean fitness of a good gene associated with a mating advantage, *W*_good gene_ (*m*, *s* > 0), is less than the mean fitness of a gene for paternal care with a similar mating advantage, *W*_good parenting_ (*m*, c_♂_ > 0). Nonetheless, a good gene with a mating advantage evolves more rapidly than a gene for good parenting with a similar mating advantage (Figure 1). This difference between mean fitness and allele frequency change occurs because the viability effect of the good gene is transmitted by both parents, while the paternal care allele is limited to males.

### 3.3 Paternal care can evolve even when it trades off with male mating success

**FIGURE 2:**
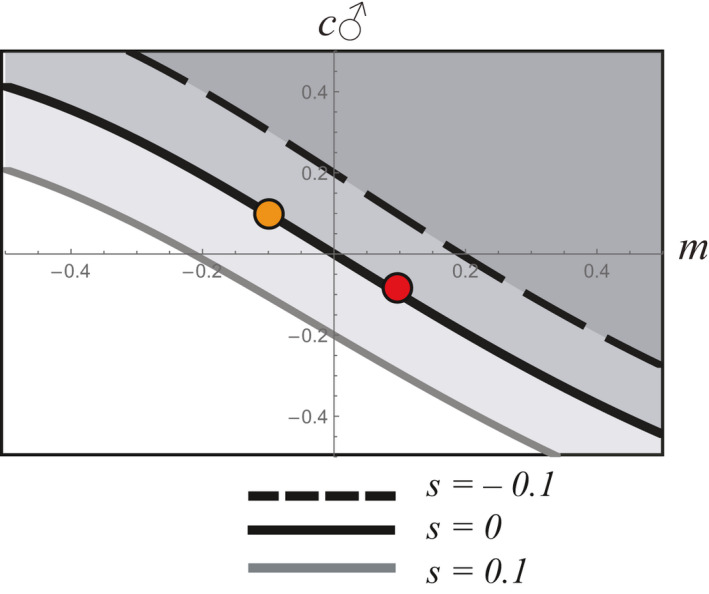
The evolutionary fate of an allele with pleiotropic effects on mating success, paternal care, and viability where each line represents the boundary between two regions: one region where the allele is favored and evolves (darker shading) and one region where the allele is not favored and does not evolve (lighter shading). In the absence of a direct effect on viability (solid line), the boundary is determined by a 1‐to‐1 trade‐off between male mating advantage and male caregiving. For example, if average viability of the population were 0.7, then a paternal care allele that increased offspring viability by 0.07 (10%) would spread even if it lowered male mating success, as long as that reduction was by less than 10% (orange dot). Conversely, an allele causing males to harm their offspring, reducing their viability by 0.07, could spread if harming males can take away mates from other males 10% or more of the time (red dot). When the direct effect on viability is negative, the boundary between regions in which the allele does and does not evolve (dashed line) is still determined by a 1‐to‐1 trade‐off (same slope as solid line), but the effects of paternal care or mating success must be substantially larger in order for the allele to overcome the associated viability cost and evolve nonetheless (dark gray region). Conversely, an allele with a positive direct effect on viability (light gray line) can potentially spread even if it significantly reduces both paternal care and male mating success (light gray region)

Next, we examine one of the conditions that is considered the primary obstacle to the evolution of paternal care: the trade‐off with a mating advantage. Here, the two episodes of selection oppose one another, and by setting *m* < 0 and c_♂_ > 0, we can investigate the potential for the indirect genetic effect of male care to evolve in spite of an associated cost to male mating success. Despite such a trade‐off, we find significant potential for paternal care to evolve (Figure 2; upper left quadrant). Similarly, we can examine the inverse situation by setting *m* > 0 > c_♂_ such that some male genotypes enjoy a mating advantage despite harming their offspring or the ability of their mate to care for offspring (Figure 2; lower right quadrant). The direct effect of viability facilitates or constrains the evolution of paternal care, depending on the direction of the association.

## 4 EXTRAPAIR COPULATIONS

We now introduce the effects of extrapair copulations (EPCs). The predominant hypothesis for EPCs is that they are an adaptive strategy of one sex or the other (Arnqvist & Kirkpatrick, 2005; Westneat & Stewart, 2003) and therefore are expected to be nonrandom. However, because the evidence for nonrandom EPCs is equivocal (Brommer et al., 2007), we model the effects of random and nonrandom EPCs on the evolution of paternal care. Surveys indicate that EPCs occur in more than 90% of socially monogamous bird species and, among studies that have used molecular markers to discern paternity, the average frequency of EPY is 11.1%, although it can be much higher in some species than in others or at some generations than others within the same species (Griffith et al., 2002). In our model, this multispecies, multi‐generation average rate of EPY corresponds to an *e* value of 0.111. In total, we examine three scenarios that include EPCs: (1) random mating followed by random EPCs; (2) random mating followed by nonrandom EPCs; and (3) nonrandom mating followed by random EPCs. We exclude analysis of nonrandom mating followed by nonrandom EPCs because such a scenario simply exaggerates the initial effects of nonrandom mating. We make no a priori assumptions about whether EPCs are the result of male or female strategies; our model can represent either scenario.

### 4.1 Random mating followed by random extrapair copulations

We start by modeling two successful bouts of random mating. After randomly mating once, we let EPCs occur with a second male, also at random, and assume that these EPCs result in extrapair young (EPY). As a result, on average, each nest consists of a fraction, (1 − *e*), of young sired by the first male and a fraction, *e*, of EPY sired by a second male (Table 2) so that whenever *e* > 0, the increment of genetic diversity added to the average clutch by EPY is (*epq*/4) and, on average, a caring male raises some offspring sired by another male. (Note, with two sires, the genetic variance within a family increases from (*pq*/2) to (*pq*/4). When *e* > 0, there are a fraction, *e*, of such families.)

**TABLE 2:** After mating randomly, females subsequently seek extrapair copulations resulting in a fraction, *e*, of extrapair young (EPYs)

Male	Female	Family frequency	Offspring genotypes			Family mean fitness
			CC	Cc	cc	
CC	CC	P^2^	1(1 − *e*) p_♂_ *e*	– q_♂_ *e*	–	1 + q_♂_ *es*
CC	Cc	PH	½(1 − *e*) p_♂_ *e*/2	½(1 − *e*) *e*/2	– q_♂_ *e*/2	1 + *s*(1 + 2 q_♂_ *e*)/2
CC	cc	PQ	–	1(1 − *e*) p_♂_ *e*	– q′*e*	1 + *s*(1 + q_♂_ *e*)
Cc	CC	HP	½(1 − *e*) p_♂_ *e*	½(1 − *e*) q_♂_ *e*	–	1 + *s*(1 – *e*[p_♂_ − q_♂_)/2 + 1c_♂_
Cc	Cc	H^2^	¼(1 − *e*) p_♂_ *e*/2	½ (1 − *e*) *e*/2	¼(1 − *e*) q_♂_ *e*/2	1 + *s*(2‐*e*[p_♂_−q_♂_])/2 + 1c_♂_
Cc	cc	HQ	–	½(1 − *e*) p_♂_ *e*	½(1 − *e*) q_♂_ *e*	1 + 1c_♂_ + *s*(3−*e*[p_♂_−q_♂_])/2
cc	CC	QP	– p_♂_ *e*	1(1 − *e*) q_♂_ *e*	–	1 + *s*(1 − *ep*) + 2c_♂_
cc	Cc	QH	– p_♂_ *e*/2	½(1 − *e*) *e*/2	½(1 − *e*) q_♂_ *e*/2	1 + 3s/2 – sep_♂_ + 2c_♂_
cc	cc	Q^2^	–	– p_♂_ *e*	1(1 − *e*) q_♂_ *e*	1 + 2c_♂_ + *s*(2 – *e* p_♂_)

Note

When the EPCs are random, q_♀_ = q_♂_ and p_♀_ = p_♂_. When the EPCs are nonrandom then the q_♂_ is the frequency of the c allele in offspring of extrapair males, while q is its frequency in females. When *m* > 0, then q_♀_ < q_♂_; when *m* < 0, q_♀_ > q_♂_; and, when *m* = 0, that is, EPCs are random, q_♀_ = q_♂_. And, *W* = 1 + 2*q*(*s* + c_♂_) + *se*(p_♀_ q_♂_ – p_♂_q_♀_) for all *e*. Note that, with random mating, the last term is 0 because p_♀_ q_♂_ = p_♂_q_♀_.

*W* = 1 + 2*s* + 2c_♂_ for all *e*.

With the entries in Table 2, we find that *W*, mean fitness, equals {1 + 2*q*(*s* + c_♂_) + *se*(p_♀_ q_♂_ − p_♂_ q_♀_)}, for all *e*. Note that, with random mating and random EPYs, the last term is 0 because p_♀_q_♂_ = p_♂_ q_♀_ = *pq*. Thus, random EPCs do not affect mean fitness. Although EPCs do redistribute a portion of the offspring viability effects of paternal care, *random EPCs* do not do so *differentially* with respect to offspring genotype. Instead, they cause a fraction of paternal care, *e*, to be *randomly* distributed over all offspring genotypes, while a fraction, (1 − *e*), remains differentially experienced by a care‐giving male's own offspring. As a result, the adaptive effect of paternal care is reduced by random EPCs from (c_♂_/2) to [(1 − *e*)c_♂_/2]). Thus, to the same degree of approximation as in the earlier case of no EPCs, we find.

(11) $$\Delta q∼\left\{\left(s+\left[\left(1‐e\right)c_{♂}/2\right]\right)pq\right\}/W.$$

It is clear that the effect of the random EPCs is to reduce the regression of offspring family mean on paternal genotype from (1/2), when *e* = 0, to (1 − *e*)/2, when *e* > 0. In the limit, if males were to raise and care for offspring entirely at random (i.e., *e* = 1), the regression of offspring mean on the paternal genotype would be 0 and there would be no effect of male parental care on gene frequency change, although it would still have an effect on mean offspring viability fitness, W (i.e., 2*q*c_♂_). *Thus, random EPCs change the weightings of paternal care in Δq but not in W*. By comparison, there is no effect of random EPCs on either *W* or Δ*q* with female care, because the regression of offspring mean on maternal genotype remains 1/2, for all 0 ≤ *e* ≤ 1, as long as a female lays all of her eggs (both those sired by her social mate and those by her extrapair mates) in the nest that she delivers care to (Wade, 1998, 2001).

**FIGURE 3:**
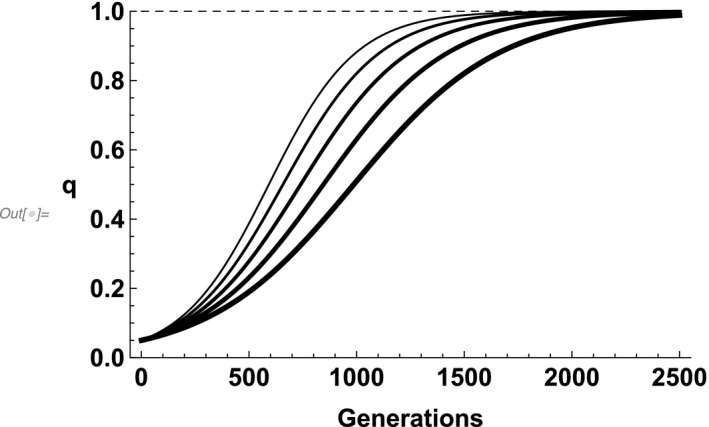
The relative rate of evolution, using exact equations, for paternal care with random mating followed by random EPCs. Direct effects of c are absent in all scenarios (i.e., *s* = 0), indirect effect of paternal care are present and identical in all cases (c = 0.01), and e increases with line width (e = 0, 0.1, 0.2, 0.3, 0.4 respectively). Dashed horizontal line represents fixation and paternal care allele starts at frequencies of 0.05)

We note that, as expected, EPCs (i.e., *e* > 0) result in a sex difference in the strength of selection for parental care: With EPCs, where c_♀_ = c_♂_, selection is always stronger for care provided by females than it is for care provided by males. However, with an average value of *e* equal to 0.11, the selection coefficients favoring the evolution of parental care in females and males are 0.50c_♀_ and 0.45c_♂_, respectively. When c_♀_ = c_♂_, this is a relatively small sex difference in selection, resulting in a Δ*q* > 0 and, consequently, selection for male parental care. We illustrate this in Figure 3, which shows that, with random EPCs, a gene for care evolves even with a relatively high frequency of EPCs. (This result changes when we introduce a male mating bias that trades off with paternal care [see below].)

### 4.2 Random mating followed by nonrandom EPCs (results in Figure 4)

If EPCs are an adaptive behavior, extrapair matings may be nonrandom with respect to male genotype (Brommer et al., 2007; Westneat & Stewart, 2003). Females may seek EPCs to increase genetic diversity of their brood, to avoid deleterious or obtain good genes, to mitigate inbreeding depression (Pilakouta et al., 2016), to obtain complementary genes, paternal offspring care, or a mating advantage for sons. The pattern of random mating followed by nonrandom EPCs can occur when the opportunity for an initial choice of mate is absent or restricted, so that females express their mating preferences only in EPCs, a pattern called “trading up” (Zeh & Zeh, 2003). Male behaviors can also result in EPCs (Arnqvist & Kirkpatrick, 2005). Male genotypes that continue courting and mating after an initial bout random mating are the type of nonrandom EPCs we model here.

**FIGURE 4:**
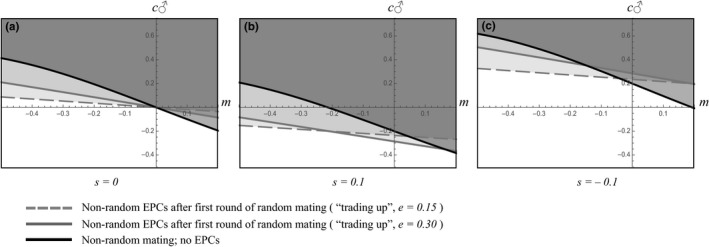
The effects of nonrandom extrapair copulation, after one round of random mating, on the evolution of paternal care. Relative to nonrandom mating without EPCs (solid black line), the parameter space in which paternal care can evolve is greater when nonrandom EPCs follow random mating in the natural range (e = 0.30, solid gray line; e = 0.15, dashed gray line). This overall pattern remains whether (a) direct effects of viability are absent, (b) direct effects of viability correlate with paternal care, or (c) direct effects of viability trade‐off with paternal care

After an initial bout of random mating, we apply the mating advantage model developed above. Since a fraction, (1 − *e*), of the matings are random, followed by a fraction, *e*, that are nonrandom, the change in q_♂_ is reduced from Δ*q_m_*/2 to *e*Δ*q_m_*/2. This reduces the selective effect of both the mating advantage (from *m* to em) and the indirect effect of paternal care (from c_♂_ to (1 − *e*) c_♂_). However, whenever *e* < 0.5, EPCs reduce the selective effect of paternal care *less than* they reduce the selective effect of a male mating advantage. Consequently, and in contrast to intuition, paternal care can evolve even in the face of relatively high levels of nonrandom EPCs and even when care‐giving trades off with a mating advantage (c > 0, *m* < 0). In fact, when EPCs take place among up to half the males (*e* < 0.5), the presence of EPCs *increases* the parameter space over which paternal care can evolve (Figure 4a–c). This general pattern remains true even when care‐giving males not only forfeit mating opportunities but also have reduced viability (Figure 4c).

### 4.3 Nonrandom mating followed by random EPCs

An initial bout of nonrandom mating followed by random EPCs has an effect different from that where the mating‐type order is reversed. And, the magnitude of the effect depends upon the gene frequency in the pool of males from which the EPCs are drawn. When mated females seek random EPCs from the pool of all males, including those male genotypes less successful at mating initially, then the change in q_♂_ from the mating bias is reduced from Δ*q_m_*/2 to (1 − *e*)Δ*q_m_*/2. Females might do this to increase the genetic diversity within their clutch. This reduction in the effect of differential mating equals the EPC reduction of brood paternity, reducing c_♂_ to (1 – *e*) c_♂_. In this case, EPCs do not *differentially* affect the relative strengths of male mating advantage and paternal care. Instead, EPCs reduce *both* effects relative to *s*, the direct effect of the “c” allele on offspring viability. On the other hand, when mated females seek random EPCs only from the pool of already mated males, then the mating‐selection change in q_♂_ remains Δ*q_m_*/2 while the EPCs reduce of c_♂_ to (1 − *e*) c_♂_ as above.

## 5 DISCUSSION

We modeled the evolution of a single gene with pleiotropic effects on three fitness components, male mating bias (*m*), paternal care (or harm, c_♂_), and offspring viability (*s*). This allows us to evaluate trade‐offs and synergies among these fitness components within a common, formal framework. Our core findings are these. First, our model reiterates an important formal point that is often overlooked; the weightings of direct and indirect fitness effects are different for mean fitness than they are for gene frequency change (Wolf & Wade, 2016). However, when a male mating advantage is introduced, it *differentially* changes the influence of paternal care and good genes (see Equation 5). That is, nonrandom mating has twice as large an effect on paternal care through c_♂_ as it has on viability through *s*. Nevertheless, when we then compare a male mating bias for a good viability gene with a mating bias of the same magnitude for good paternal care, we find a surprising result. Although a mating bias for a good viability gene raises mean fitness by a smaller amount than an equivalent mating bias for good paternal care, a gene for good viability nevertheless evolves more rapidly than a gene for good paternal care. This comparison illustrates with an example that caution should be used when drawing evolutionary conclusions from fitness optimization arguments.

Next, our study shows that paternal care can evolve even if it comes at the expense of male mating success. If the indirect effect of paternal care is sufficient, it can even overcome a trade‐off with both reduced male mating success and reduced viability in both sexes. Additional support for this theoretical finding comes from studies that find evidence for paternal care even in species where it almost certainly represents opportunity costs for males (e.g., (Buchan et al., 2003; Safari et al., 2019). However, the extent to which such a trade‐off is found across species remains an open question (Stiver & Alonzo, 2009), and in some cases, male parental behaviors may evolve that avoid or mitigate associated costs. For instance, in some species courtship and mating are temporally distinct from the period in which juveniles benefit from care. Thus, males that provide care may not have reduced mating success, and in such cases, the evolution of paternal care should not be considered a puzzle at all.

Finally, the evolution of paternal care can occur despite EPCs, at least at the high end of the observed range. Indeed, some scenarios for EPCs, such as “trading up,” even *facilitate* the evolution of paternal care. We showed that, when there is a trade‐off between paternal care and male mating advantage, precare EPCs affect the strength of that trade‐off. They can skew it toward paternal care (increasing its influence on evolutionary change) and away from male mating advantage (diminishing its influence on evolutionary change). In other words, neither the forgoing of mating opportunities nor EPCs preclude the evolution of paternal care. Our theoretical findings support and formalize suggestions that paternal care and paternity uncertainty can co‐exist (Klug et al., 2012; Sheldon, 2002). Our findings are also supported by a cross‐taxa analysis of avian species, which found no relationship between the frequency of EPCs and many types of paternal care (Møller & Birkhead, 1993). Indeed, within one population of house wrens, the degree of paternal care (provisioning rates) is *positively* correlated with the number of extrapair young (LaBarbera et al., 2012).

Discussions of the evolution of paternal care, female mate choice, good genes, and extrapair copulations frequently hypothesize that “direct” fitness costs of paternal care can be offset by “indirect” fitness benefits, such as mating ability of sons or increased survivorship of offspring (Arnqvist & Kirkpatrick, 2005; MØller & Thornhill, 1998). Whenever there is a trade‐off, the fitness benefits of paternal care must outweigh the cost of foregoing fertilizations for it to evolve. However, our model reveals that evaluating these trade‐offs is complicated by two factors. First, the impact of one fitness component on mean fitness is not equivalent to its impact on gene frequency change. And, second, changing one fitness component or the timing of EPCs *differentially* alters the evolutionary consequences of the other fitness components on both mean fitness and gene frequency change. Our analysis suggests that paternal care can readily evolve under several conditions and point to specific scenarios that are ripe for empirical study.

## CONFLICTS OF INTEREST

There authors declare no conflicts of interest.

## AUTHOR CONTRIBUTION

**Courtney Fitzpatrick:** Conceptualization (equal); Formal analysis (supporting); Investigation (equal); Visualization (lead); Writing‐original draft (lead); Writing‐review & editing (lead). **Colette M. Ciresi:** Conceptualization (equal); Formal analysis (supporting); Investigation (equal); Writing‐review & editing (supporting). **Michael J. Wade:** Conceptualization (equal); Formal analysis (lead); Investigation (equal); Visualization (supporting); Writing‐original draft (lead); Writing‐review & editing (lead).

## Data Availability

Mathematica files for equations and simulations: Dryad https://doi.org/10.5061/dryad.xwdbrv1c8
